# Antibiotic quality and use practices amongst dairy farmers and drug retailers in central Kenyan highlands

**DOI:** 10.1038/s41598-023-50325-8

**Published:** 2023-12-28

**Authors:** Dishon M. Muloi, Peter Kurui, Garima Sharma, Linnet Ochieng, Fredrick Nganga, Fredrick Gudda, John Maingi Muthini, Delia Grace, Michel Dione, Arshnee Moodley, Caroline Muneri

**Affiliations:** 1 Animal and Human Health Department, International Livestock Research Institute, Nairobi, Kenya; 2 Department of Veterinary Surgery, Theriogenology and Medicine, Egerton University, Njoro, Kenya; 3 Department of Biochemistry, Microbiology and Biotechnology, Kenyatta University, Nairobi, Kenya; 4 Natural Resources Institute, University of Greenwich, Kent, UK; 5 Animal and Human Health Department, International Livestock Research Institute, Dakar, Senegal; 6 Department of Veterinary and Animal Sciences, University of Copenhagen, Frederiksberg C, Denmark; 7 Institute of Infection, Veterinary and Ecological Sciences, University of Liverpool, Neston, UK

**Keywords:** Antimicrobial resistance, Epidemiology

## Abstract

Understanding antibiotic use in dairy systems is critical to guide antimicrobial stewardship programs. We investigated antibiotic use practices in small-holder dairy farms, antibiotic quality, and antimicrobial resistance (AMR) awareness among veterinary drug retailers in a mixed farming community in the central Kenyan highlands. Data were collected from 248 dairy farms and 72 veterinary drug stores between February 2020 and October 2021. A scale was developed to measure knowledge about AMR and antibiotic use using item response theory, and regression models were used to evaluate factors associated with antibiotic use and AMR knowledge. The active pharmaceutical ingredient (API) content of 27 antibiotic samples was determined using high-performance liquid chromatography (HPLC). The presence and levels of 11 antibiotic residues in 108 milk samples collected from the study farms were also investigated using liquid chromatography tandem mass spectrometry (LC–MS/MS). Almost all farms (98.8%, n = 244) reported using antibiotics at least once in the last year, mostly for therapeutic reasons (35.5%). The most used antibiotics were tetracycline (30.6%), penicillin (16.7%), and sulfonamide (9.4%), either individually or in combination, and predominantly in the injectable form. Larger farm size (OR = 1.02, p < 0.001) and history of vaccination use (OR = 1.17, p < 0.001) were significantly associated with a higher frequency of antibiotic use. Drug retailers who advised on animal treatments had a significantly higher mean knowledge scores than those who only sold drugs. We found that 44.4% (12/27) of the tested antibiotics did not meet the United States Pharmacopeial test specifications (percentage of label claim). We detected nine antibiotics in milk, including oxytetracycline, sulfamethoxazole, and trimethoprim. However, only three samples exceeded the maximum residue limits set by the Codex Alimentarius Commission. Our findings indicate that antibiotics of poor quality are accessible and used in small-holder dairy systems, which can be found in milk. These results will aid future investigations on how to promote sustainable antibiotic use practices in dairy systems.

## Introduction

Animal husbandry currently accounts for approximately two thirds of the global consumption of antibiotics, and this is projected to increase^1^. The widespread use and misuse of antibiotics has raised concerns about potential development and dissemination of antimicrobial resistance (AMR). AMR infections have been estimated to lead to 1.27 million deaths globally in 2019, with most of the burden borne by low- and middle-income countries (LMICs), particularly in sub-Saharan Africa^2^. In these settings, antibiotics serve as ‘quick fixes’ for hygiene and productivity challenges, acting as substitutes for more costly interventions to improve the conditions within which health workers, farming communities, and animals work and live^3^. In the recent years, there are increasing calls for reduction in the use of antibiotics in the agri-food system^4^, with a growing emphasis on the importance of antimicrobial stewardship (AMS) and good animal husbandry practices^5^. In Kenya, as other countries, the government has developed a National Action Plan on AMR (AMR NAP), which aligns with the global AMR action plan, and prioritizes antimicrobial stewardship in livestock systems^6^.

Kenya's dairy sector, predominantly consists of smallholder farmers, owning between one to three cows, and contributes a substantial portion to the country's Gross Domestic Product (GDP) derived from livestock farming (14% of the agricultural GDP and 3.5% to the overall GDP)^7,8^. Use of antibiotics is widespread in these dairy systems, as they are plagued with mastitis, respiratory infections, enteric diseases; and “dry cow” therapy is a predominant reason for antibiotic use^9^. The extensive use of antibiotics raises concerns about the excretion of antibiotic residues in milk, which can pose health risks to humans and ecosystems^10^ and development of AMR in human and bovine pathogens. However, few studies have investigated how these antibiotics are accessed and used by farmers, levels of antibiotic residues in milk, and knowledge and practices related to on-farm antibiotic use.

Furthermore, most smallholder farmers are not able to afford veterinary services, which may also be scarce depending on the region, thus leading them to either self-diagnose or rely on veterinary drug stores for consultation, diagnosis, and prescription of drugs, including antibiotics^11,12^. However, irrational and inappropriate prescription of antibiotics is a common practice in these stores because most staff are not qualified to diagnose and prescribe treatment, some are legally not permitted to prescribe^11,13^, and sales of antibiotics are linked to profits for the veterinary drug stores business^14^. The situation is further exacerbated by the high reported prevalence of counterfeit and substandard veterinary medicines in Sub Saharan Africa^15^. A recent systematic review reported that 47.3% of medicines tested in 17 African countries were either substandard or falsified^16^. In a similar study from the Mekong Delta in Vietnam, 6.9% of the antibiotic sampled in veterinary drug stores contained levels of active pharmaceutical ingredients (APIs) outside the pharmacopeial limits^17^.

This study reports the results of a cross-sectional survey in dairy farms and veterinary drug stores in a Kenyan mixed farming community to assess (1) on-farm antibiotic usage patterns and practices, (2) determine the levels of antibiotic residues in milk, (3) prescription practices amongst veterinary drug stores workers, and (4) determine the quality of selected antibiotics purchased from veterinary drug stores.

## Methods

### Study area and population

The study population was a crop-livestock mixed farming community in Kericho county located in the highlands of the Kenyan Rift Valley. Kericho County is characterized by high levels of rainfall and agricultural productivity, and most households grow a range of crops for subsistence and to sell. Dairy farming, with an average herd size of < 5 cows, is often integrated with crop farming, mainly tea and maize production, on small plots of 1–5 acres^18^.

All research reported here was performed in accordance with the Declaration of Helsinki and the legal requirements of the Government of Kenya. Ethical approval for human data collection was obtained from The International Livestock Research Institute (ILRI) Institutional Research Ethics Committee (ILRI-IREC2020-01). Animal sampling was conducted under the approval of the ILRI Institutional Animal Care and Use Committee (ILRI-IACUC2020-06), and permits were obtained from the Directorate of Veterinary Services. Written informed consent was obtained from all study participants.

### Study design and selection of farms and veterinary drug stores

The study was cross-sectional, with the primary study unit being dairy farms in Kipkelion East, a sub-county of Kericho, while veterinary drug stores spanned the whole county. Kipkelion East sub-county was randomly selected from a list of sub-counties in Kericho. A sample size of 248 dairy farms was calculated to estimate the prevalence of antibiotic residues in milk on farms, assuming an expected prevalence of antibiotic residues 10% in milk^19^. Random selection of farms was stratified by sub-location, which is the smallest administrative unit in Kenya. Within each sub-location, farms were identified with the assistance of the local veterinary officers, as lists of farms were not available. For veterinary drug stores, a total of 72 stores were purposefully selected across the county for maximum spatial distribution. Due to the lack of a registry of veterinary drug stores, random sampling within each sub-county was not possible, but efforts were made to ensure consistent selection of study stores. The final distribution of sampled farms and veterinary drug stores is shown in Fig. S1. Data collection happened between February 2020 and October 2021.

### Data collection on dairy farms

On each farm, the farmer (or a nominated farm worker) completed a questionnaire^20,21^ detailing livestock ownership (e.g., number of dairy cows), farm management practices (e.g., history of vaccination), antimicrobial use patterns and knowledge of antibiotic use. From selected healthy milking cows on each farm, milk samples were collected from each quarter after thoroughly cleaning and drying the teats. The samples were placed immediately into a cool box with ice and refrigerated at 4 °C and transferred to the lab within five hours.

### Data collection at veterinary drug stores

A questionnaire was administered to veterinary drug store owners or workers to collect data on demographics, customer demographics, types of antibiotics sold, antibiotic prescribing practices, and knowledge of AMR and antibiotic use. In Kenya, veterinary drug stores are primarily managed by animal health technicians, also known as para-veterinarians, while only a small number are operated by veterinarians. According to the Kenyan regulations, animal health technicians are not authorized to prescribe antibiotics, only veterinarians. In this study, the term “pharmacist” refers to individuals involved in the sale of antibiotics, regardless of their level of clinical training.

Concurrently with the questionnaire survey, a mystery shopper, assumed the role of a farmer and visited veterinary drug stores, presenting two scenarios: Scenario 1 involved a dairy cow experiencing respiratory distress symptoms such as nasal discharge, lethargy, labored breathing, and lack of appetite; Scenario 2 involved a flock of broilers displaying symptoms of diarrhea, weight loss, weakness, lack of appetite, and ruffled feathers. One of the shoppers, who was a veterinarian, further requested a specific antibiotic brand. The mystery shoppers were not denied an antibiotic despite not having a prescription. The samples were purchased in their original containers, labelled with unique numbers, and the following information was collected on each sample: brand name, active pharmaceutical ingredients, manufacturer's name, package size, strength, and dosage form. Data from the questionnaires were recorded using Open DataKit (ODK) Collect software on electronic tablets and uploaded to databases at the International Livestock Research Institute (ILRI), Nairobi, Kenya.

### Laboratory analysis of antibiotic residues in milk

Details on sample extraction and testing are provided in the supplementary methods, but briefly, we used ultra-high performance liquid chromatography-tandem mass spectrometry (LC–MS/MS) to determine the concentration of antibiotic residues in milk. The LC–MS/MS experiments were performed using the Shimadzu LC–MS 8050 system (Shimadzu Corporation, Kyoto, Japan) consisting of LC-20AD solvent delivery pumps, a SIL-30AC autosampler, a CTO-30A column oven and the LC–MS 8050 triple quadrupole detectors. Detection by MS/MS was performed on a Shimadzu 8050 triple quadrupole mass spectrometer, fitted with an electrospray ionization source operating in positive ionization mode. Samples were tested for tetracycline, oxytetracycline, chlortetracycline, penicillin G, sulfamethoxazole, trimethoprim, gentamicin, ceftiofur, dihydrostreptomycin, ampicillin, and chloramphenicol. Antibiotic levels in milk were compared to Codex Alimentarius Commission maximum residue limits for veterinary drugs^22^.

### Laboratory analysis of veterinary drugs

Details on sample preparation and testing are provided in the supplementary methods, but briefly, we used a validated LC–MS/MS method on a Shimadzu SPD-20A Prominence Diode Array (PDA) UV–VIS Detector DAD HPLC system to measure the active pharmaceutical ingredient (API) content in each sample. Duplicate homogeneous mixtures of the samples were prepared and analysed, with the analysis conducted blindly, without prior knowledge of the brand. The assessment of drug quality was based on the mean percentage of API per label and compliance with the United States Pharmacopeial 29 (USP 29) standard on content assay (percentage content) for the respective antibiotics. Samples that exceeded these limits were classified as non-compliant and further divided into two categories based on the extent of deviation from the USP 29 criteria: moderate deviations and extreme deviations (Table s1).

### Modelling drivers of antibiotic use in dairy farms

Data analysis was performed using R. Descriptive analysis displayed frequencies and percentages for categorical variables and mean, standard deviation, and range for quantitative variables. Univariable analyses were performed to test for independent associations between the various predictor variables and the frequency of antibiotic use on dairy farms. The outcome of interest was the number of times antibiotics used on farm during the year before the study. The explanatory factors included history of vaccination, total number of livestock on the farm, utilization of laboratory services, participation in animal health campaigns, seeking professional veterinary help, frequency of milk sales, grazing type, participation in farmer trainings, utilization of household waste as feed, and use of commercial premixed feed. Variables with a p-value < 0.2 were included in a multivariable Poisson generalized linear model (GLM) to identify significant (p < 0.05) explanatory variables associated with farm-level antibiotic use. The multivariable models were fitted using the lme4 package in R, and significance was determined using Wald χ2-tests. The best-fitting model was selected based on the lowest Akaike Information Criterion (AIC) using the 'dredge' function in the MuMIn package^23^. Diagnostics plots of the models were generated using the DHARMa package^24^ to assess whether the model assumptions were violated.

### Analysis of knowledge of antibiotics and antibiotic use amongst veterinary drug store retailers

Information on knowledge and practices related to AMR and antibiotic use were collected from veterinary pharmacists using a 3-point Likert scale (‘agree,’ ‘disagree,’ and ‘don't know’). The responses were dichotomized, with ‘agree’ recoded as one (1) and ‘disagree’ or ‘don't know’ as zero (0). Cronbach’s alpha was calculated to assess reliability, with a value greater than 0.70 indicating good consistency of item responses. The unidimensionality of a scale based on the knowledge questions was evaluated using confirmatory factor analysis (CFA) using the *cfa function* in lavaan package^25^. A two-parameter logistic item response theory (IRT) model was applied using the ltm^26^ package to estimate the latent level of knowledge among respondents. Four items with low discriminatory power were removed from the analysis. The remaining ten items were used to create item characteristic curves (ICC) and item information functions (IIF) to evaluate the measurement scale's performance. Knowledge scores for each respondent were derived using empirical Bayes predictions using *factor.scores* function in ltm^26^. A generalized linear model was used to examine the relationship between veterinary drug shop demographics and prescription practices with the latent knowledge score.

## Results

### Demographic characteristics of study participants

Out of the 248 dairy farmers who participated in the survey, 145 (58.5%) were men, and their average age was 46.7 years. The mean herd size was 7.4 (range, 0–40), with almost all farmers (96.8%) practicing zero grazing. Private animal health providers (AHPs), either paraveterinarians or veterinarians, served as the primary veterinary care providers (98%) for farmers in this study. A third of farmers had participated in animal health campaigns within the last year, while a quarter of them had attended trainings specifically focused on disease prevention.

A total of 72 veterinary drug stores were surveyed. More than two thirds (70.8%) of the veterinary drug store pharmacists were male, with a mean age of 34.1 years (range 19–60). Of the pharmacists surveyed, 45.8% were exclusively involved in retail business, while 54.2% were engaged in both retailing and private veterinary practice. Additionally, 23.6% had less than one year of experience, 24% had 1–5 years of experience, and 31% had more than five years of experience. Meanwhile, 25% of the respondents did not have animal health training but only completed secondary or primary school.

### Antibiotic access and use amongst dairy farmers

**Figure 1:**
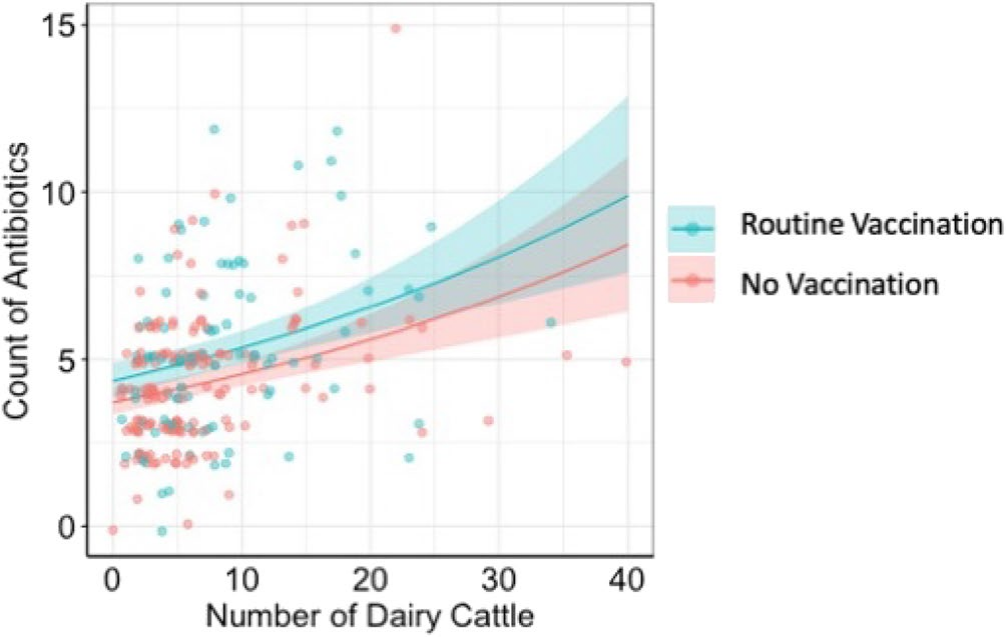
Fit of the Poisson generalized linear model relating the number of dairy cattle on study farms, vaccination history, and number of antibiotics used. The 95% confidence interval is represented by the colored bands.

Of the 248 dairy farms surveyed, 245 (98.8%) used antibiotics at least once in the last year. Moreover, 35.5% of farmers reported only using antibiotics for therapeutic reasons, 4.9% used prophylactic reasons, and 5.6% for both purposes. Tetracycline (30.6%, n = 75), penicillin (16.7%, n = 41), and sulfonamide (9.4%, n = 23) were the most frequently used antibiotic classes, either individually or in combinations, and predominantly in the injectable form. Other antibiotics reported include aminoglycosides (6.1%, n = 15), macrolides (0.8%, n = 2), and fluoroquinolones (0.4%, n = 1). Most farmers (90.3%) reported buying antibiotics from animal health service providers, while 5.7% bought them directly from veterinary drug stores, and the remaining farmers sourced antibiotics from other farmers. Univariable analysis revealed significant associations between antibiotic use and several factors, including number of livestock on farm, vaccination status, utilization of laboratory services in the last year, participation in farmer training programs, frequency of milk sales, and the practice of using household waste as animal feed (Table S2). The multivariable analyses revealed that larger farms and those with history of vaccination use were significantly associated with a higher frequency of antibiotic use (OR = 1.02, p < 0.001, 95%CI [1.01–1.03]; OR = 1.17, p < 0.001, 95%CI [1.04–1.31], respectively) (Fig. 1).

Dairy farmers were the most frequent customers (77.8%) who purchased antibiotics in the stores followed by poultry farmers (22%). One-third of veterinary drug stores described antibiotics as the product that contributed the most to their sales, while only one store (1.4%) mentioned vaccines as their primary product. Eighty six percent of stores reported that they sold antibiotics without a prescription, relying on drug labels and the information provided by the farmers for making a diagnosis and deciding on treatment choices including the prescribing of antibiotics.

### Antibiotic residues in milk

A total of 108 milk samples collected from farms and analyzed for nine antibiotics. All nine antibiotics were detected in varying rates above the detection limit with oxytetracycline (50%, n = 54), sulfamethoxazole (50%, n = 54) and trimethoprim (40.7%, n = 44) detected in more than a third of the samples (Table S4). However, only three samples, containing tetracycline (0.9%), oxytetracycline (2.8%), and penicilin G (4.6%) were above their respective MRLs.

### Knowledge of AMR and antibiotic use amongst veterinary pharmacists

**Figure 2:**
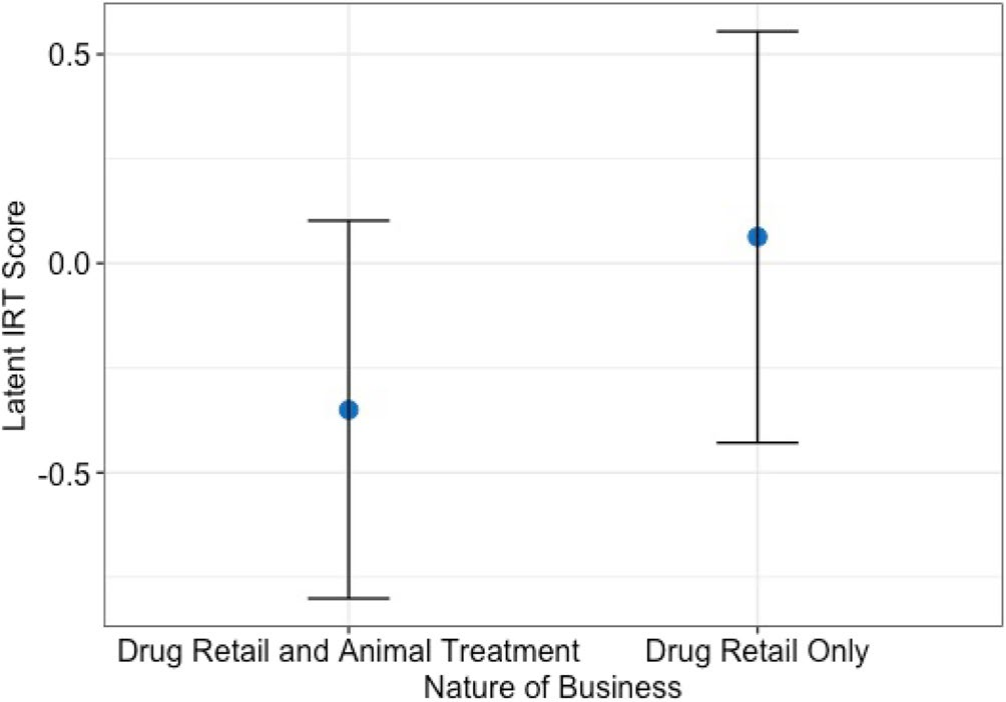
Fit of the generalized linear model relating the knowledge scale and demographic attributes. The 95% confidence interval is represented by the error bars.

The internal reliability of the ten “knowledge statements” was Cronbach’s α of 0·72. Most of the questions had a similar discrimination level and difficulty level expect for Q8 which had a higher difficulty of 1.07 and Q5 which had a discrimination level of 2.35 (Table S3 and Fig. S2). Our final model indicated that respondents working in drug retail businesses who also advised on animal treatments had a significantly higher mean for knowledge than those who only sold drug (p = 0.03, GLM, Fig. 2). There was no significant difference between having knowledge and whether prescriptions are required or not, respondents age, gender, education level or duration in business (p > 0.05, GLM).

### Quality of antibiotics purchased from veterinary drug stores

**Figure 3:**
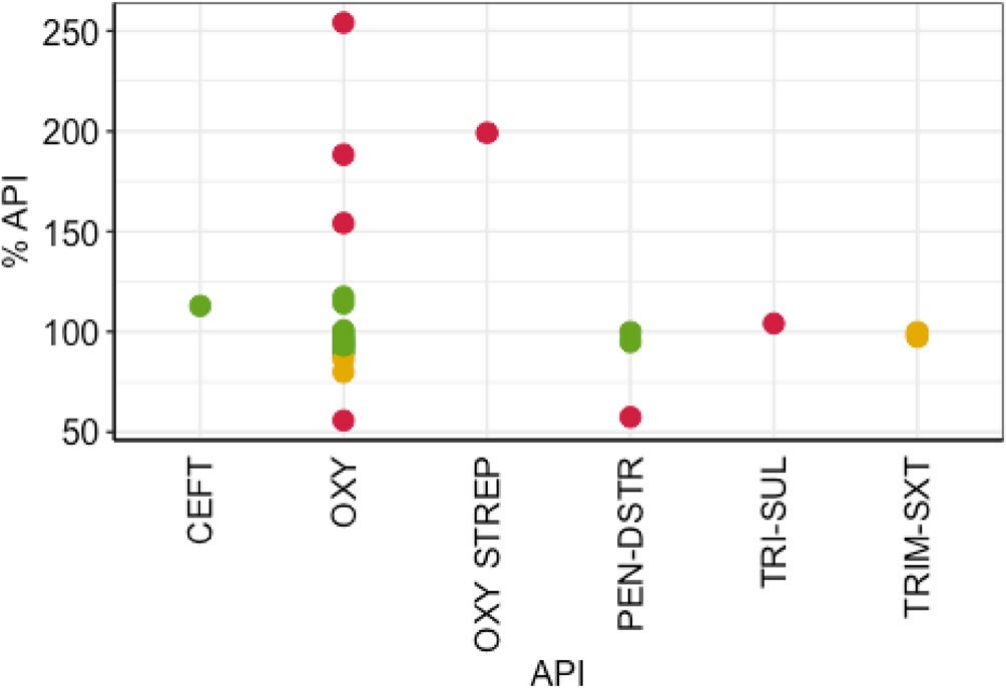
Content of the active pharmaceutical ingredient (API) determined for each antibiotic purchased. Each sample is represented by a dot, with red color indicating extreme deviation, amber indicating moderate deviation, and green indicating compliance within the quality range. EFT, ceftiofur; OXY, oxytetracycline; DSTR, dihydrostreptomycin; PEN, penicillin; SXT, sulfamethoxazole; TMP, trimethoprim; TET, tetracycline; SUL, sulfadiazine; STR, streptomycin.

A total of 27 antibiotics were purchased and of those, 19 (70.3%) were single compound antibiotics and the remaining eight (29.6%) were combinations of two or more antibiotics. Oxytetracyline (66.7%, 18), was the most frequently sold antibiotic. The other antibiotics include sulfamethoxazole/trimethoprim (11.1%, 3), sulfadimidine/trimethoprim (3.7%, 1), penicillin G/dihydrostreptomycin (11.1%, 3), cefitour (3.7%, 1), and oxytetracycline/streptomycin (3.7%, 1). Of the 19 samples with one antibiotic, 13 (65%) were within the API-specific limit ranges of USP29 (% label claim), four (20%) showed extreme deviations and two (15%) showed moderate deviations. Of the eight antibiotic combination samples, two (25%) were within acceptable range, and remaining six (75%) failed (Fig. 3 and Table s4).

## Discussion

This study aimed to describe practices associated with antibiotic use in dairy farms and antibiotic sale and quality of antibiotics in veterinary drug stores within a mixed farming community in central Kenyan highlands. This study extends previous research on non-prescription antibiotic sales at veterinary drug stores in LMICs^11,13,27^. Despite legislation prohibiting sales of antibiotics without a prescription in Kenya^28^, this practice remains common, and is driven by factors such as animal health services either too costly and inaccessible for farmers, weak enforcement of regulations by the government, and financial motivations amongst pharmacists. Improving pharmacists’ knowledge and practice of antimicrobial stewardship has been posited as a possible and cost-effective strategy to reduce non-prescription antibiotic sale^29^. Similarly, veterinary drug stores should be encouraged to diversify their business to sell other products such as vaccines, vitamins, and provide veterinary services, which would provide an alternative source of income and reduce reliance on antibiotic sales^30^. However, we found that knowledge of antibiotics or AMR did not vary by level of animal health training, nor did it influence whether a pharmacist sold an antibiotic with or without a prescription. There is an increasing agreement that antimicrobial stewardship education programs alone may not be enough to sustain improvements in optimized antibiotic use^31,32^. Instead, these programs should be integrated with reinforced legislation, strong surveillance systems along the antibiotic supply chain, and consumer education for behavioural change.

Most dairy farmers in this study reported using antibiotics, mainly for therapeutic purposes, with higher usage observed in larger farms and those implementing routine vaccinations. Our finding that most farmers relied on animal health service providers for animal health management, which placed the responsibility of antibiotic stewardship on them, highlights the complexity of on-farm antibiotic decision-making processes. Animal health service providers have minimal access to diagnostic testing to support treatment (antibiotic) choice, and the definition of “normal” use is not well-defined. Targets that define acceptable amounts of antibiotics to be used and metrics of quantitative values of antibiotic used in farming systems are critically needed for AMS, but these are lacking in LMIC settings. We found antibiotic residues in milk that exceeded the MRLs for the three antibiotics, tetracycline, oxytetracycline and penicillin G. This finding is consistent with previous research documenting similar results in milk collected along the milk value chain^33–35^, and is reflective of imprudent antibiotic use practices particularly non-adherence to antibiotic withdrawal guidelines. To guide risk assessment and the development of evidence-based animal health policies, antibiotic use, and residue monitoring surveillance programs should be considered^36^. Antibiotic use was associated with herd size suggesting that poor animal management increase the likelihood of infections requiring antibiotic treatment. However, history of vaccination did not reduce the likelihood of antibiotic use, despite the widely accepted knowledge that the use of vaccines in livestock substantially decreases antibiotic use^37^. This discrepancy raises the possibility that farms that vaccinated their animals modified their behavior based on the perception of being protected, potentially neglecting adequate biosecurity measures that reduce infections and subsequent antibiotic use. Another possible explanation is potentially low vaccine efficacy, possibly due to poor handling along the supply chain, the use of the wrong vaccine, or poor administration^38^.

Despite anecdotal evidence suggesting that poor-quality veterinary antibiotics are common in Sub-Saharan Africa, prevalence data is lacking^16,39,40^. The presence of poor-quality antibiotics poses significant risks to animal health and welfare, food and nutritional safety, farmers’ profits and raising concerns about the potential emergence and spread of AMR^41,42^. In our study, we found that 44.4% (12/27) of the tested antibiotics did not meet the United States pharmacopeial test specifications for assay (% label claim). These findings align with previous research conducted in Vietnam, where 6.9% of antibiotic were found to have concentrations less than half of the labeled content^17^. The underlying reasons for the presence of poor-quality antibiotics include poor manufacturing practices, falsification of contents, poor pharmacovigilance, degradation during the supply chain, and inadequate storage conditions^43,44^. We could not test these hypotheses using our data; however, a combination of epidemiological surveys with robust methodology, larger sample sizes, and chemical analyses including MS fingerprinting may help distinguish among these factors^45^. A multi-pronged approach combining regulatory improvements that focus on pharmacovigilance, strengthening laboratory capacity, and increasing public awareness about the importance of using high-quality antibiotics are needed to enhance health of antibiotic supply chains.

The limitations of this study are inherent to the epidemiological methods used in our cross-sectional survey, which focused on describing antibiotic sales, antibiotic use, and antibiotic quality in a specific rural dairy farming community. Therefore, generalization of the findings to urban populations, where these practices may differ, is limited. Additionally, the data obtained from veterinary drugs stores relied on information provided by pharmacy staff and was not cross validated with veterinary or sales records. Furthermore, the analysis of antibiotic quality was based on a sample size of 27 samples, and storage conditions or periods in the veterinary store could have influenced our findings.

## Conclusion

We characterized antibiotic sale, use, and quality patterns in a dairy farming community in Kenya. Our findings indicate that antibiotics, often of poor quality, are frequently sold without prescription, commonly used without proper diagnostics, and can contaminate food products. With the growing threat of AMR, it is crucial for veterinary medicine stakeholders to reevaluate the role of community veterinary drug stores in antibiotic stewardship programs. In addition, we highlight the urgency to develop interventions aimed at enhancing the quality of antibiotics including stricter regulation, post-market surveillance surveys utilizing public–private partnerships, and public education.

### Supplementary Information

Supplementary Information.

## Data Availability

The datasets generated and analysed in the current study are available from the corresponding author upon reasonable request.
