# Supplementary material for: Antibiotic quality and use practices amongst dairy farmers and drug retailers in central Kenyan highlands
Source: Sci Rep. 2023 Dec 28;13:23101. doi: 10.1038/s41598-023-50325-8 (PMC10754936; doi:10.1038/s41598-023-50325-8)
Supplement: Supplementary file 1 — Supplementary Information. [file 41598_2023_50325_MOESM1_ESM.docx]

**Antibiotic quality and use practices amongst dairy farmers and drug retailers in Central Kenyan Highlands**

**Supplementary Tables**

**Table s1**. Compliance limits for the antibiotics

|  | Content of API (=assay) (% of declared content) | | |
| --- | --- | --- | --- |
| International nonproprietary names | Complies | Moderate deviation | Extreme deviation |
| Sulfamethoxazole | 93-107 | 80 to < 93 | <80 or > 120 |
| Trimethoprim | 93-107 | 80 to < 93 | <80 or > 120 |
| Oxytetracycline | 90 - 120 | 80 to < 90 | <80 or > 120 |
| Ceftiofur | 90 - 120 | 80 to < 90 | <80 or > 120 |
| Penicillin G | 90 - 120 | 80 to < 90 | <80 or > 120 |

API = Active Pharmaceutical Ingredient. The United States Pharmacopeia 29 (USP 29) specifications were used to determine the compliance limits of the samples based on the percentage of the declared API. A sample was classified in “extreme deviation” if the API content deviated by more than 20% of that declared by the manufacturer.

**Table s2.** Results of the Univariable Poisson Generalized Linear Model, illustrating the association between the number of times antibiotics were used on the farm and various risk factors.

| **Variable** | **Odds Ratio** | **95% CI** | **P-value** |
| --- | --- | --- | --- |
| Total no. of livestock | 1.99 | 1.95-2.04 | 0 |
| Vaccination status |  |  |  |
| No | Ref |  |  |
| Yes | 1.21 | 1.07-1.36 | 0 |
| Take professional help in last 12 months |  |  |  |
| No | Ref |  |  |
| Yes | 1.11 | 0.98-1.27 | 0.09 |
| Use lab services in last 12 months |  |  |  |
| No | Ref |  |  |
| Yes | 1.36 | 1.08-1.73 | 0 |
| Take part in animal health campaign in last 12 months |  |  |  |
| No | Ref |  |  |
| Yes | 1.1 | 0.98-1.24 | 0.1 |
| Attended farmer training on disease prevention/control |  |  |  |
| No | Ref |  |  |
| Yes | 1.12 | 0.99-1.28 | 0 |
| Frequency of milk sold |  |  |  |
| Throughout the year | Ref |  |  |
| Occasionally | 0.79 | 0.64-0.96 | 0.02 |
| Certain months/periods | 0.82 | 0.71-0.94 | 0 |
| Where do they sell milk |  |  |  |
| Cooperative | Ref |  |  |
| Middlemen | 0.89 | 0.76-1.05 | 0.17 |
| Neighbors | 0.76 | 0.63-0.92 | 0 |
| Others | 0.64 | 0.37-1.10 | 0.11 |
| Use household waste |  |  |  |
| No | Ref |  |  |
| Yes | 1.22 | 1.07-1.38 | 0 |
| Use commercial premixed feed |  |  |  |
| No | Ref |  |  |
| Yes | 1.11 | 0.97-1.27 | 0.12 |
| Grazing type |  |  |  |
| Fenced grazing | Ref |  |  |
| Pastoral | 0.66 | 0.43-1.00 | 0.05 |
| Tethered | 0.81 | 0.70-0.94 | 0 |
| Zero | 0.79 | 0.59-1.04 | 0.1 |

**Table s3.** IRT item parameters for the knowledge about antibiotics and AMR using the final model with problematic items removed.

| **ID** | **Statement** | **Difficultness** | **Discrimination** |
| --- | --- | --- | --- |
| Q1 | It is bacteria that can become resistant to antibiotics | -2.5929498 | 0.9194371 |
| Q2 | It is people who can become resistant to antibiotics | -0.448476 | 1.7235504 |
| Q3 | Animals can become resistant to antibiotics | 0.1518922 | 1.5905003 |
| Q4 | Antibiotic resistance is due to using antibiotics when they are not indicated | -1.7432356 | 0.9579824 |
| Q5 | Antibiotics are effective in managing bacterial infections | -1.2031288 | 2.3488472 |
| Q6 | Antibiotics are effective in managing viral infections | -0.6752297 | 1.3967901 |
| Q7 | Antibiotics are effective in managing parasites infections | -1.0019615 | 2.404159 |
| Q8 | Antibiotics are effective in managing pain and inflammation | 1.0685462 | 0.5737178 |
| Q9 | Antibiotics are effective in boosting animal growth | -0.2883777 | 0.9123403 |
| Q10 | Antibiotics residues from animals can be found in meat | -1.9952034 | 1.4963459 |

**Table s4.** Antibiotic concentration in milk samples (µg/L)

| **ID** | **SDZ** | **SMZ** | **TMP** | **TC** | **OTC** | **AMP** | **GEN** | **CTF** | **PEN G** |
| --- | --- | --- | --- | --- | --- | --- | --- | --- | --- |
| 1 | <LOD | <LOD | <LOD | <LOD | <LOD | <LOD | <LOD | <LOD | <LOD |
| 2 | <LOD | 0.74 | <LOD | <LOD | <LOD | <LOD | <LOD | <LOD | 4.79 |
| 3 | <LOD | 1.81 | <LOD | <LOD | <LOD | <LOD | <LOD | <LOD | <LOD |
| 4 | <LOD | <LOD | <LOD | <LOD | <LOD | <LOD | 9.73 | <LOD | <LOD |
| 5 | <LOD | <LOD | <LOD | <LOD | <LOD | <LOD | <LOD | <LOD | <LOD |
| 6 | <LOD | <LOD | <LOD | <LOD | <LOD | <LOD | 28.15 | <LOD | <LOD |
| 7 | <LOD | 1.38 | <LOD | <LOD | <LOD | <LOD | <LOD | <LOD | <LOD |
| 8 | <LOD | 1.47 | <LOD | <LOD | <LOD | <LOD | <LOD | <LOD | <LOD |
| 9 | <LOD | 1.45 | <LOD | <LOD | <LOD | <LOD | <LOD | <LOD | <LOD |
| 10 | 0.51 | 1.05 | <LOD | <LOD | <LOD | <LOD | <LOD | <LOD | <LOD |
| 11 | 0.50 | <LOD | <LOD | <LOD | <LOD | <LOD | <LOD | <LOD | <LOD |
| 12 | <LOD | <LOD | 0.95 | <LOD | <LOD | <LOD | <LOD | <LOD | <LOD |
| 13 | <LOD | <LOD | 0.69 | <LOD | <LOD | <LOD | <LOD | <LOD | <LOD |
| 14 | <LOD | 0.64 | 0.66 | <LOD | <LOD | <LOD | <LOD | <LOD | <LOD |
| 15 | <LOD | <LOD | 0.64 | <LOD | <LOD | <LOD | <LOD | 3.39 | <LOD |
| 16 | <LOD | 0.70 | 0.58 | <LOD | <LOD | <LOD | <LOD | <LOD | 4.78 |
| 17 | <LOD | 0.84 | 0.50 | <LOD | <LOD | <LOD | <LOD | <LOD | <LOD |
| 18 | <LOD | <LOD | 0.46 | <LOD | <LOD | <LOD | <LOD | <LOD | <LOD |
| 19 | <LOD | <LOD | 0.41 | <LOD | <LOD | <LOD | <LOD | <LOD | 4.79 |
| 20 | 1.63 | <LOD | 0.37 | <LOD | <LOD | <LOD | <LOD | <LOD | <LOD |
| 21 | <LOD | <LOD | 0.32 | <LOD | <LOD | <LOD | 11.77 | <LOD | <LOD |
| 22 | 0.50 | <LOD | 0.32 | <LOD | <LOD | <LOD | <LOD | <LOD | <LOD |
| 23 | <LOD | <LOD | 0.31 | <LOD | <LOD | <LOD | <LOD | <LOD | <LOD |
| 24 | 0.50 | <LOD | 0.27 | <LOD | <LOD | <LOD | <LOD | <LOD | <LOD |
| 25 | <LOD | 1.36 | 0.27 | <LOD | <LOD | <LOD | <LOD | <LOD | <LOD |
| 26 | <LOD | <LOD | 0.23 | <LOD | <LOD | <LOD | <LOD | <LOD | <LOD |
| 27 | <LOD | 0.78 | 0.18 | <LOD | <LOD | <LOD | <LOD | <LOD | <LOD |
| 28 | 0.28 | 1.83 | 0.41 | <LOD | <LOD | 2.14 | <LOD | <LOD | <LOD |
| 29 | <LOD | 1.71 | <LOD | <LOD | <LOD | 0.61 | <LOD | 2.03 | <LOD |
| 30 | <LOD | 1.21 | <LOD | <LOD | 37.60 | <LOD | <LOD | <LOD | <LOD |
| 31 | 0.43 | <LOD | 0.23 | <LOD | 14.76 | <LOD | <LOD | <LOD | <LOD |
| 32 | <LOD | <LOD | 0.44 | <LOD | 11.84 | <LOD | <LOD | <LOD | <LOD |
| 33 | <LOD | 1.36 | 0.23 | <LOD | 9.16 | <LOD | <LOD | <LOD | <LOD |
| 34 | <LOD | 0.78 | 0.41 | <LOD | 8.98 | <LOD | <LOD | <LOD | <LOD |
| 35 | 0.14 | 1.71 | 0.64 | <LOD | 8.03 | <LOD | <LOD | 5.44 | <LOD |
| 36 | <LOD | <LOD | 0.64 | <LOD | 7.35 | <LOD | <LOD | <LOD | <LOD |
| 37 | <LOD | 0.74 | 0.37 | <LOD | 6.12 | <LOD | 13.73 | <LOD | <LOD |
| 38 | <LOD | 1.38 | <LOD | <LOD | 6.12 | <LOD | <LOD | <LOD | <LOD |
| 39 | <LOD | 1.21 | 0.96 | <LOD | 5.71 | <LOD | <LOD | <LOD | <LOD |
| 40 | <LOD | <LOD | <LOD | <LOD | 5.30 | <LOD | <LOD | <LOD | 2.39 |
| 41 | <LOD | 0.81 | 0.49 | <LOD | 4.90 | <LOD | 15.72 | <LOD | <LOD |
| 42 | <LOD | 1.36 | <LOD | <LOD | 4.89 | <LOD | <LOD | <LOD | <LOD |
| 43 | <LOD | <LOD | <LOD | <LOD | 4.48 | <LOD | <LOD | <LOD | <LOD |
| 44 | 0.62 | 1.33 | 0.59 | <LOD | 4.08 | <LOD | <LOD | <LOD | <LOD |
| 45 | <LOD | <LOD | 0.46 | <LOD | 3.67 | <LOD | <LOD | <LOD | <LOD |
| 46 | <LOD | 1.44 | 0.18 | <LOD | 3.67 | <LOD | <LOD | <LOD | <LOD |
| 47 | <LOD | <LOD | 0.50 | <LOD | 3.26 | <LOD | 15.73 | <LOD | <LOD |
| 48 | <LOD | <LOD | 0.55 | <LOD | 3.26 | <LOD | <LOD | <LOD | <LOD |
| 49 | <LOD | 1.38 | 0.41 | <LOD | 3.24 | <LOD | 5.76 | <LOD | <LOD |
| 50 | <LOD | <LOD | <LOD | <LOD | 2.85 | <LOD | <LOD | <LOD | <LOD |
| 51 | <LOD | 0.95 | <LOD | <LOD | 2.45 | <LOD | <LOD | <LOD | <LOD |
| 52 | 0.28 | <LOD | 0.37 | <LOD | 2.44 | <LOD | <LOD | <LOD | <LOD |
| 53 | <LOD | <LOD | <LOD | <LOD | 2.04 | <LOD | <LOD | <LOD | <LOD |
| 54 | <LOD | 1.28 | 0.50 | <LOD | 2.04 | <LOD | <LOD | <LOD | <LOD |
| 55 | <LOD | 1.12 | 0.59 | <LOD | 2.04 | <LOD | <LOD | <LOD | <LOD |
| 56 | <LOD | 1.26 | 0.50 | <LOD | 1.63 | <LOD | <LOD | <LOD | <LOD |
| 57 | <LOD | <LOD | 0.18 | <LOD | 1.63 | <LOD | <LOD | <LOD | <LOD |
| 58 | <LOD | 1.80 | 0.46 | <LOD | 1.63 | <LOD | <LOD | <LOD | <LOD |
| 59 | <LOD | <LOD | 0.32 | <LOD | 1.63 | <LOD | <LOD | <LOD | <LOD |
| 60 | <LOD | 1.38 | 0.32 | <LOD | 1.62 | <LOD | <LOD | <LOD | <LOD |
| 61 | <LOD | 0.72 | 0.81 | <LOD | 1.22 | <LOD | <LOD | <LOD | <LOD |
| 62 | <LOD | 2.02 | 0.37 | <LOD | 1.22 | <LOD | <LOD | <LOD | <LOD |
| 63 | 1.63 | 1.78 | 0.37 | <LOD | 1.22 | <LOD | <LOD | <LOD | <LOD |
| 64 | <LOD | <LOD | 0.28 | <LOD | 1.22 | <LOD | <LOD | <LOD | <LOD |
| 65 | 0.28 | <LOD | <LOD | <LOD | 1.22 | <LOD | <LOD | <LOD | <LOD |
| 66 | <LOD | <LOD | 0.55 | <LOD | 1.22 | <LOD | <LOD | <LOD | <LOD |
| 67 | <LOD | <LOD | 0.18 | <LOD | 1.22 | <LOD | <LOD | <LOD | <LOD |
| 68 | <LOD | <LOD | 0.37 | <LOD | 1.22 | <LOD | <LOD | <LOD | <LOD |
| 69 | 0.26 | 0.73 | <LOD | <LOD | 0.81 | <LOD | <LOD | <LOD | <LOD |
| 70 | 42.69 | 2.33 | 38.37 | <LOD | 0.81 | <LOD | <LOD | <LOD | <LOD |
| 71 | 0.49 | <LOD | 0.41 | <LOD | 0.81 | <LOD | <LOD | <LOD | <LOD |
| 72 | 0.25 | 0.96 | <LOD | <LOD | 0.81 | <LOD | <LOD | <LOD | <LOD |
| 73 | <LOD | <LOD | <LOD | <LOD | 0.81 | <LOD | <LOD | <LOD | <LOD |
| 74 | <LOD | 0.42 | 0.68 | <LOD | 0.40 | <LOD | <LOD | <LOD | <LOD |
| 75 | <LOD | <LOD | 0.55 | <LOD | 0.40 | <LOD | <LOD | <LOD | <LOD |
| 76 | 0.20 | 2.21 | 1.85 | 177.00 | 623.03 | 1.22 | 25.62 | <LOD | <LOD |
| 77 | 1.13 | 3.17 | <LOD | 70.63 | <LOD | 1.22 | 51.26 | <LOD | <LOD |
| 78 | <LOD | 4.41 | <LOD | 29.30 | 47.74 | <LOD | <LOD | <LOD | <LOD |
| 79 | <LOD | 2.05 | 2.33 | 17.61 | 27.78 | <LOD | <LOD | <LOD | <LOD |
| 80 | 0.35 | 1.74 | 0.78 | 11.06 | 46.38 | <LOD | <LOD | <LOD | <LOD |
| 81 | <LOD | 3.18 | 1.19 | 9.37 | <LOD | <LOD | <LOD | 0.67 | <LOD |
| 82 | <LOD | 0.53 | 0.78 | 9.14 | 9.39 | <LOD | <LOD | <LOD | <LOD |
| 83 | 4.31 | 0.54 | 3.92 | 8.66 | 99.61 | <LOD | <LOD | <LOD | <LOD |
| 84 | 0.42 | <LOD | 0.46 | 8.18 | 8.58 | <LOD | <LOD | <LOD | <LOD |
| 85 | <LOD | <LOD | 0.32 | 6.74 | 219.71 | <LOD | <LOD | <LOD | <LOD |
| 86 | 0.37 | 1.08 | <LOD | 6.26 | <LOD | <LOD | <LOD | <LOD | <LOD |
| 87 | <LOD | <LOD | 0.47 | 6.25 | 11.43 | <LOD | <LOD | <LOD | <LOD |
| 88 | 0.14 | 1.45 | <LOD | 5.82 | 54.94 | <LOD | <LOD | 2.71 | <LOD |
| 89 | <LOD | 2.45 | 6.45 | 5.77 | 101.13 | <LOD | <LOD | <LOD | <LOD |
| 90 | <LOD | <LOD | 0.73 | 4.94 | 5.50 | <LOD | <LOD | <LOD | <LOD |
| 91 | <LOD | 1.67 | 0.50 | 4.81 | <LOD | <LOD | <LOD | <LOD | <LOD |
| 92 | 0.57 | <LOD | 0.35 | 4.81 | 6.12 | <LOD | <LOD | <LOD | <LOD |
| 93 | 0.89 | <LOD | 1.26 | 4.32 | 10.21 | <LOD | <LOD | <LOD | <LOD |
| 94 | <LOD | 0.42 | <LOD | 4.32 | 53.58 | <LOD | <LOD | <LOD | 20.41 |
| 95 | <LOD | <LOD | <LOD | 3.83 | 9.80 | <LOD | <LOD | <LOD | <LOD |
| 96 | <LOD | 2.33 | 0.59 | 2.88 | 2.04 | <LOD | <LOD | <LOD | <LOD |
| 97 | <LOD | 0.90 | 0.73 | 1.92 | 2.85 | <LOD | <LOD | <LOD | <LOD |
| 98 | <LOD | <LOD | <LOD | 1.92 | 1.62 | <LOD | <LOD | <LOD | <LOD |
| 99 | 0.14 | <LOD | 0.09 | 1.92 | 0.40 | <LOD | <LOD | <LOD | <LOD |
| 100 | <LOD | 1.17 | 0.14 | 1.91 | 2.85 | <LOD | <LOD | <LOD | <LOD |
| 101 | 0.75 | <LOD | 0.39 | 1.44 | 4.08 | <LOD | <LOD | <LOD | <LOD |
| 102 | <LOD | 1.51 | 0.31 | 0.96 | 0.81 | <LOD | <LOD | <LOD | <LOD |
| 103 | <LOD | 1.02 | 0.46 | 0.96 | <LOD | <LOD | <LOD | <LOD | <LOD |
| 104 | <LOD | <LOD | 0.32 | 0.96 | <LOD | <LOD | <LOD | <LOD | 4.79 |
| 105 | <LOD | 1.12 | 0.72 | 0.95 | <LOD | 2.76 | <LOD | <LOD | <LOD |
| 106 | <LOD | 2.12 | 0.78 | 0.95 | 3.27 | <LOD | <LOD | <LOD | <LOD |
| 107 | <LOD | <LOD | 0.45 | 0.47 | <LOD | <LOD | <LOD | <LOD | <LOD |
| 108 | 0.28 | 0.89 | 0.95 | 0.47 | 7.75 | <LOD | <LOD | <LOD | <LOD |

SMZ: Sulfamethoxazole, SDZ: Sulfadiazine, TMP: Trimethoprim, TET: Tetracycline, OTC: Oxytetracycline, AMP: Ampicillin, GEN: Gentamicin, CTF: Ceftiofur, PEN G: Penicillin G. Limits for LOD and LOQ are provided in Table S6.

**Table s5.** Description of the antibiotics sampled in the survey.

| **Sample ID** | **Active Ingredient (AI)** | **Injection (mg/ml)** | **Mean conc.** | **% API** |
| --- | --- | --- | --- | --- |
| 011B | Oxytetracycline | 0.04 | 0.039 | 96.67 |
| 020A | Oxytetracycline | 0.04 | 0.04 | 100.83 |
| 002B | Oxytetracycline | 0.04 | 0.038 | 95.83 |
| 018B | Oxytetracycline | 0.04 | 0.039 | 97.5 |
| 001B | Oxytetracycline | 0.04 | 0.046 | 114.17 |
| 022B | Oxytetracycline | 0.04 | 0.04 | 100 |
| 005B | Oxytetracycline | 0.04 | 0.036 | 90 |
| 007B | Oxytetracycline | 0.04 | 0.036 | 90 |
| 035B | Oxytetracycline | 0.04 | 0.035 | 86.25 |
| 046B | Oxytetracycline | 0.04 | 0.022 | 55.83 |
| 031B | Oxytetracycline | 0.04 | 0.032 | 80 |
| 006B | Oxytetracycline | 0.04 | 0.033 | 93.33 |
| 048B | Oxytetracycline | 0.04 | 0.037 | 93.33 |
| 008A | Oxytetracycline | 0.04 | 0.037 | 93.33 |
| 012B | Penicillin G | 0.02 | 0.019 | 95 |
|  | Dihydrostreptomycin | 0.04 | - | - |
| 019B | Penicillin G | 0.02 | 0.02 | 100 |
|  | Dihydrostreptomycin | 0.04 | - | - |
| 009B | Penicillin G | 0.02 | 0.012 | 57.5 |
|  | Dihydrostreptomycin | 0.04 | - | - |
| 055B | Trimethoprim | 0.04 | 0.042 | 104.17 |
|  | Sulfadimidine | Not tested |  |  |
| 056B | Ceftiofur | 0.08 | 0.09 | 112.92 |
| 026B | Trimethoprim | 0.04 | 0.04 | 100 |
|  | Sulfamethoxazole | 0.2 | 0.194 | 96.83 |
| 030B | Trimethoprim | 0.04 | 0.039 | 97.5 |
|  | Sulfamethoxazole | 0.2 | 0.178 | 89 |
| 003B | Trimethoprim | 0.04 | 0.039 | 98.33 |
|  | Sulfamethoxazole | 0.2 | 0.164 | 82.17 |
| 025B | Oxytetracycline | 0.04 | 0.08 | 199.17 |
| 027B | Oxytetracycline | 0.04 | 0.061 | 154.17 |
| 032B | Oxytetracycline | 0.04 | 0.075 | 188.33 |
| 015B | Oxytetracycline | 0.04 | 0.047 | 117.5 |
| 049B | Oxytetracycline | 0.04 | 0.102 | 254.17 |

Symbol ‘-‘ represents a failed test. The "% API" column is color-coded according to pharmacopeial compliance, where red represents extreme deviation, amber indicates moderate deviation, and green signifies compliance within the limit range.

**Supplementary Figures**


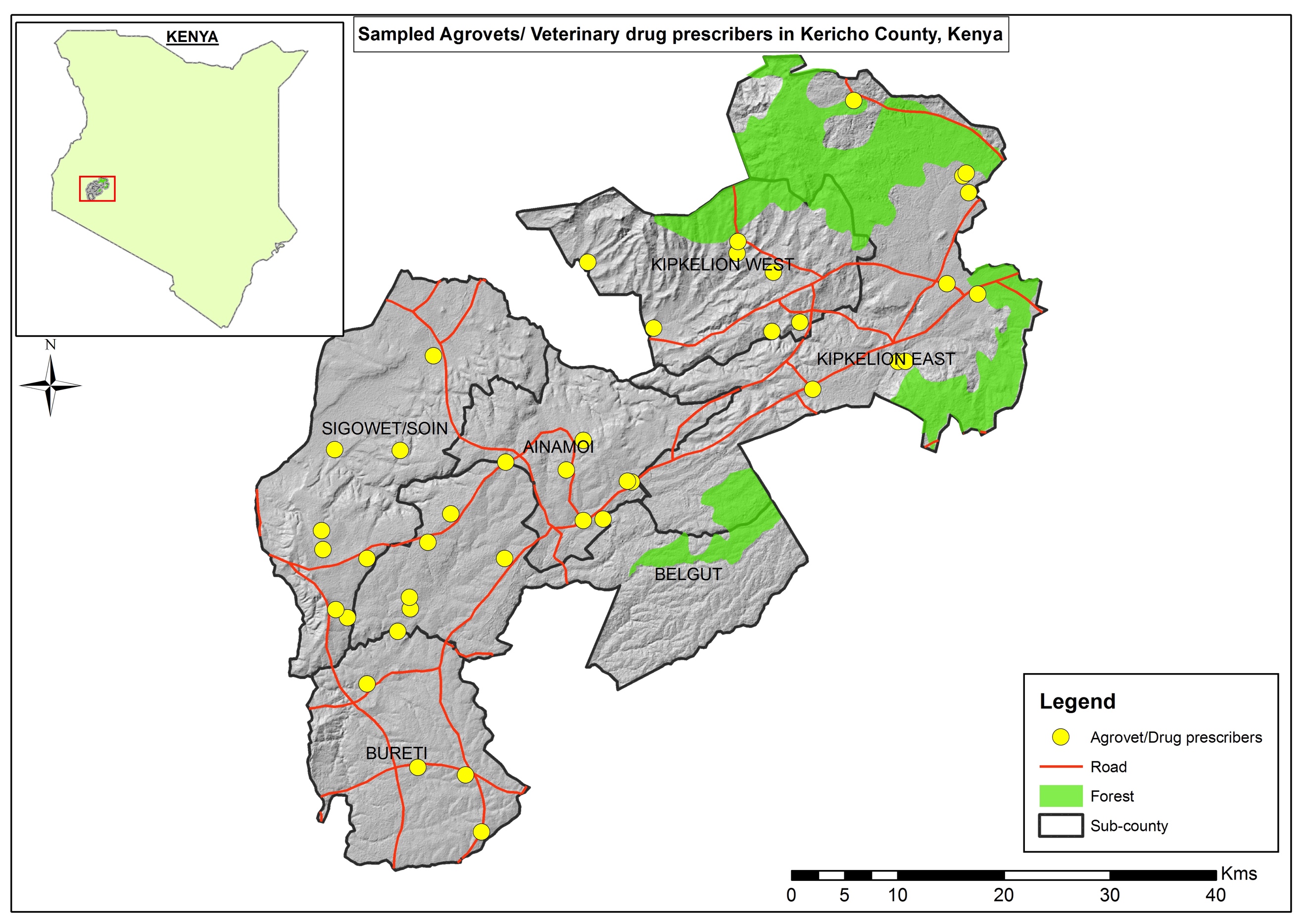

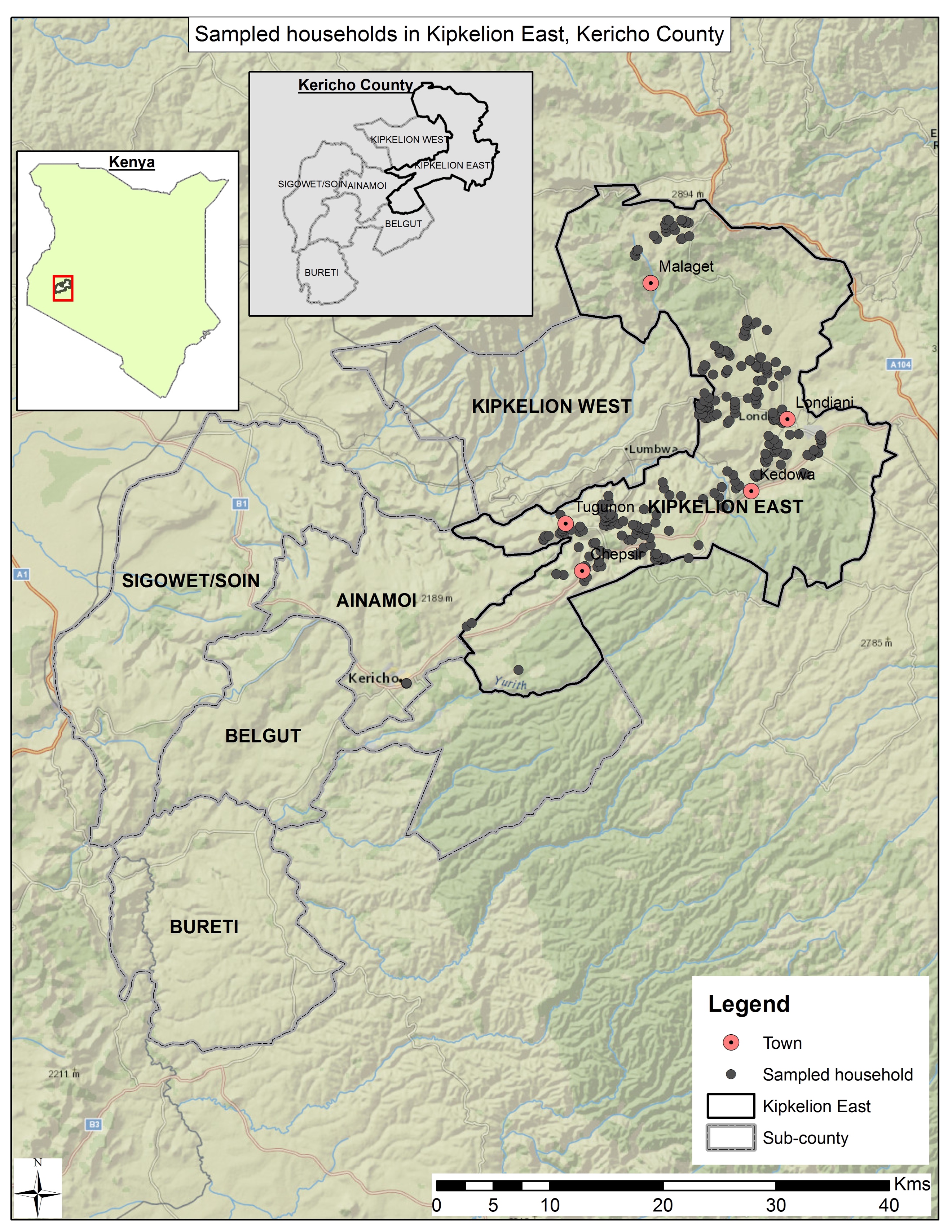


A

B

**Figure s1.** Map of Kericho county showing the A) Farms and B) Veterinary drug stores sampled during the survey.


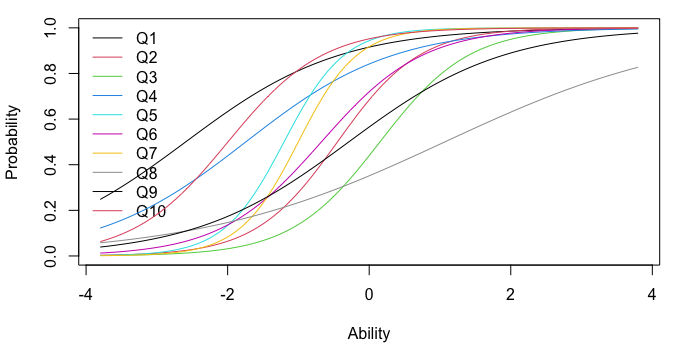


**Figure s2.** Item characteristic curves for the final model. (Items: Q1 It is bacteria that can become resistant to antibiotics; Q2 It is people who can become resistant to antibiotics; Q3 Animals can become resistant to antibiotics; Q4 Antibiotic resistance is due to using antibiotics when they are not indicated; Q5 Antibiotics are effective in managing bacterial infections ; Q6 Antibiotics are effective in managing viral infections; Q7 Antibiotics are effective in managing parasites infections; Q8 Antibiotics are effective in managing pain and inflammation; Q9 Antibiotics are effective in boosting animal growth; Q10 Antibiotics residues from animals can be found in meat).

**Supplementary methods:**

Sample processing and analysis for both veterinary drug residues and active pharmaceutical ingredients were carried out at the Mycotoxin and Nutritional Analysis Platform of the International Livestock Research Institute (ILRI) in Nairobi, Kenya.

1. **Laboratory analysis of residues in milk**
2. **Chemicals and reagents**

The following chemicals and reagents were sourced from Sigma Aldrich (St. Louis, MO, USA) for the analysis: Chromasolv-grade methanol (MeOH), acetonitrile (ACN), formic acid, ammonium acetate, ethylenediaminetetraacetic acid (EDTA), dimethyl sulfoxide (DMSO), and antibiotic residue standards including sulfadiazine, sulfamethoxazole, trimethoprim, tetracycline, oxytetracycline, ampicillin, gentamycin, ceftiofur, and Penicillin G.

1. **Sample extraction**

The extraction of antibiotic residues was performed following the method described by Sosienski[1]s. Briefly, 2 grams of milk were centrifuged in a 50ml centrifuge tube. Then, 400 μL of 0.5M EDTA solution was added and shaken for 5 minutes. Afterward, 7.6 ml of a solution containing 2% formic acid and 2% dimethyl sulfoxide in acetonitrile was added to the sample and shaken for an additional 5 minutes. The solution was then centrifuged at 4500 rpm for 5 minutes and 1 ml of the resulting solution filtered through a 0.2 μm filter into amber-colored HPLC vials.

1. **Sample analysis via LC-MS-MS**

The identification and quantification of antibiotic residues were performed using the Shimadzu Nexera liquid chromatograph system coupled to the LC–MS/MS 8050 triple quadrupole mass spectrometer detector (Shimadzu Corporation, Kyoto, Japan). This system comprised the SIL-30AC autosampler, LC-20AD solvent delivery pump, CTO-30A column oven, and 8050 triple quadrupole mass spectrometry detector. Separation was done using a Kinetex 2.6u C18, 100 x 3.00 mm column (Phenomenex, Torrance, CA, USA) at a temperature of 40°C with a flow rate of 0.4 mL/min. A binary mobile phase system was used, consisting of mobile phase A (0.2% formic acid, 99:1, v/v) and mobile phase B (0.5 mM ammonium acetate in methanol).

A gradient elution program was set with the following conditions: 0 minutes, 2% mobile phase B; at 2.5 minutes, 70% mobile phase B; at 5 minutes, 100% mobile phase B; at 10 minutes, 2% mobile phase B. The system was then held for an additional 2 minutes for re-equilibration, resulting in a total run time of 12 minutes. Next, detection of the analytes was performed using the Shimadzu 8050 triple quadrupole mass spectrometer with an electrospray ionization source operating in both positive and negative ionization modes. The ionization source was configured with nebulizing gas and drying gas flow rates of 3 L/min and 10 L/min, respectively; interface voltage set to 4.5 kV; desolvation line temperature maintained at 300 ◦C, and the heating block temperature set to 400 ◦C. Direct infusion of individual antibiotic neat standards into the mass spectrometer was performed to identify the transitions in multiple reaction monitoring (MRM) mode for quantitation. Optimized MRM parameters and the corresponding antibiotic retention time are shown in Table s5.

Table s5. LC-MS/MS optimized multiple reaction monitoring (MRM) parameters for analytes

| Antibiotic | Retention time (sec) | Ionisation mode | Molecular weight | Precursor ion | Product ion | Q1 Pre-bias | Collision energy | Q3 Pre-Bias |
| --- | --- | --- | --- | --- | --- | --- | --- | --- |
| Sulfadiazine | 3.27 | ESI+ | 250.278 | 251.28 [M+H]^+^ | 108.15/92.15/155.95 | -14.0/-13.0/-14.0 | -23.0/-27.0/-15.0 | -22.0/-17.0/-28.0 |
| Sulfamethoxazole | 3.47 | ESI+ | 253.279 | 254.28 [M+H]^+^ | 92.10/107.95 | -14.0/-15.0/-19.0 | -26.0/-46.0/-23.0 | -18.0/-27.0/-21.0 |
| oxytetracycline | 3.38 | ESI+ | 460.434 | 461.00 [M+H]^+^ | -14.0/-14.0/-25.0 | -20.0/-14.0/ -36.0 | -20.0/-14.0/-36.0 | -21.0/-22.0/-22.0 |
| Tetracycline | 3.36 | ESI+ | 444.435 | 445.15 [M+H]^+^ | -24.0/-24.0/-14.0 | -24.0/-24.0/-14.0 | -19.0/-13.0/-40.0 | -30.0/-21.0/-22.0 |
| Trimethoprim | 3.29 | ESI+ | 290.32 | 291.33 [M+H]^+^ | 230.15/261.15/123.10 | -23.0/-11.0/-24.0 | -23.0/-25.0/-26.0 | -25.0/-29.0/-21.0 |
| Ceftiofur | 3.61 | ESI+ | 523.56 | 524.10 [M+H]^+^ | 241.10/121.95/209.95 | -20.0/-20.0-20.0 | -18.0/-50.0/-21.0 | -26.0/-26.0/-26.0 |
| Ampicillin | 3.94 | ESI+ | 349.406 | 350.10 [M+H]^+^ | 106.0/192.05/114.100 | -19.0/-10.0/-11.0 | -20.0/-16.0/-31.0 | -21.0/-20.0/-23.0 |
| Gentamycin | 3.49 | ESI+ | 477.596 | 479.10 [M+H]^+^ | 462.10/444.00/154.00 | -15.0/-27.0/-15.0 | -19.0/-23.0/-30.0 | -23.0/-22.0/-29.0 |
| Penicillin G | 3.78 | ESI+ | 334.4 | 335.10 [M+H]^+^ | 160.15/176.10/114.15 | -18.0/-18.0/-18.0 | -13.0/-14.0/-33.0 | -11.0/-19.0/-23.0 |

Method validation was performed according to Magnusson and Örnemark [2] , encompassing parameters such as recovery, linearity, limit of detection (LOD), limit of quantification (LOQ), and investigating the signal suppression/enhancement ratio. The results of the validation, including data on these parameters, can be found in Table s6.

Table S6. LC-MS/MS method validation and performance characteristics: linearity, spike recovery, matrix effects, and analytical limits

| Antibiotic | Spike levels (ng/g) | Average recovery (%) | RSD (%) | Signal suppression enhancement (SSE) % | Coefficient of correlation  R^2^ | LOD  (ng/g) | LOQ  (ng/g) |
| --- | --- | --- | --- | --- | --- | --- | --- |
| sulfadiazine | 25,50,100,200 | 88.03 | 2.41 | 56.43 | 0.9993 | 0.34 | 1.23 |
| Sulfamethoxazole | 25,50,100,200 | 81.13 | 2.60 | 74.28 | 0.9998 | 0.18 | 0.68 |
| oxytetracycline | 25,50,100,200 | 75.67 | 5.34 | 119.08 | 0.993 | 0.41 | 1.50 |
| Tetracycline | 25,50,100,200 | 80.54 | 6.08 | 103.16 | 0.9957 | 0.29 | 1.07 |
| Trimethoprim | 25,50,100,200 | 108.89 | 2.35 | 100.45 | 0.9966 | 0.12 | 0.423 |
| Ceftiofur | 25,50,100,200 | 72.02 | 3.53 | 40.93 | 0.9992 | 0.70 | 2.55 |
| Ampicillin | 25,50,100,200 | 68.12 | 5.66 | 113.24 | 0.9991 | 0.55 | 2.00 |
| Gentamycin | 25,50,100,200 | 103.59 | 5.77 | 59.23 | 0.9996 | 0.27 | 0.97 |
| Penicillin G | 25,50,100,200 | 69.84 | 8.18 | 54.97 | 0.9997 | 0.56 | 2.06 |

Quantification was done using external calibration with matrix-matched calibration standards, employing 10µl injections. Antibiotic levels in milk were compared to the maximum residue limits for veterinary drugs established by the Codex Alimentarius Commission.

1. **Laboratory analysis of active pharmaceutical ingredient**
2. **Sample Preparation**

100mg of the pharmaceutical formulation was dissolved or diluted with 10ml of absolute methanol. This solution was then subjected to two subsequent dilutions: first, a 1:15 (v/v) dilution, followed by a final 1:10 (v/v) dilution, resulting in a total dilution of 1:150 (v/v). Next, 0.5ml of the diluted sample was added to 1.5ml amber-colored UPLC vials, preparing the extract for HPLC analysis. Calibration standards for oxytetracycline, Penicillin G, Trimethoprim, Ceftiofur, and sulfamethoxazole were prepared in methanol, covering a range of 1-40 µg/l.

**Instrumental analysis**

Chromatographic separation was performed using the Shimadzu Nexera X2 HPLC system, which consisted of a Sil 30AC autosampler, LC-20AD solvent delivery pump, CTO-30A column oven, and SPD-20A Prominence Diode Array detector. A Kinetex 2.6u C18, 100 x 3.00 mm column from (Phenomenex, Torrance, CA, USA) was utilized, operating at a flow rate of 0.3 mL/min. The column oven temperature was set at 35°C. A binary mobile phase system was employed, consisting of methanol as mobile phase A and water as mobile phase B. An isocratic gradient elution program with 60% mobile phase B was utilized, which provided satisfactory peak resolution for the antibiotic molecules. Injection volumes of 10 µL were used for both the standards and sample extracts. Data on the performance of the chromatographic method, including retention time, linearity, and λmax, is shown in Table s7. observed HPLC method performance characteristics for active pharmaceutical ingredient analysis).

Table s7. Observed HPLC method performance characteristics for active pharmaceutical ingredient analysis.

| Antibiotic | Retention time (min) | λ_max (nm)_ | Coefficient of correlation  R^2^ |
| --- | --- | --- | --- |
| Oxytetracycline | 1.94 | 360 | 0.9931 |
| Penicilin G | 3.201 | 293 | 0.9979 |
| Trimethoprim | 3.325 | 286 | 0.9976 |
| Ceftiofur | 1.933 | 290 | 0.9969 |
| Sulfamethoxazole | 2.33 | 260 | 0.997 |

The assessment of drug quality was conducted by evaluating the percentage of active pharmaceutical ingredient (API) per label and comparing it to the United States Pharmacopeial 29 (USP 29) standard on content assay (percentage content) for the specific antibiotics. Samples that exceeded these limits were classified as non-compliant. Non-compliant samples were further categorized into two groups based on the degree of deviation from the USP 29 criteria: moderate deviations and extreme deviations (Table S1).

References:

1. Sosienski, T., *Veterinary Drug Detection in Pork and MilkUsing an Ultivo LC/TQ with a standard ESI ion source*. 2018, Agilent Technologies.

2. Bertil, M. and U. Örnemark, *The fitness for purpose of analytical methods: a laboratory guide to method validation and related topics.* A laboratory guide to method validation and related topics, LGC, Teddington, Middlesex, UK, 2014.
